# An Amperometric Biosensor Based on a Bilayer of Electrodeposited Graphene Oxide and Co-Crosslinked Tyrosinase for L-Dopa Detection in Untreated Human Plasma

**DOI:** 10.3390/molecules28135239

**Published:** 2023-07-06

**Authors:** Giuseppa Cembalo, Rosanna Ciriello, Carmen Tesoro, Antonio Guerrieri, Giuliana Bianco, Filomena Lelario, Maria Assunta Acquavia, Angela Di Capua

**Affiliations:** Dipartimento di Scienze, University of Basilicata, Via dell’Ateneo Lucano 10, 85100 Potenza, Italy; giuseppacembalo@gmail.com (G.C.); carmen.tesoro@unibas.it (C.T.); antonio.guerrieri@unibas.it (A.G.); giuliana.bianco@unibas.it (G.B.); filomena.lelario@unibas.it (F.L.); angela.dicapua@unibas.it (A.D.C.)

**Keywords:** L-Dopa, electrochemical detection, tyrosinase, co-crosslinking, graphene oxide, electrodeposition, human plasma

## Abstract

L-Dopa, a bioactive compound naturally occurring in some Leguminosae plants, is the most effective symptomatic drug treatment for Parkinson’s disease. During disease progression, fluctuations in L-DOPA plasma levels occur, causing motor complications. Sensing devices capable of rapidly monitoring drug levels would allow adjusting L-Dopa dosing, improving therapeutic outcomes. A novel amperometric biosensor for L-Dopa detection is described, based on tyrosinase co-crosslinked onto a graphene oxide layer produced through electrodeposition. Careful optimization of the enzyme immobilization procedure permitted to improve the long-term stability while substantially shortening and simplifying the biosensor fabrication. The effectiveness of the immobilization protocol combined with the enhanced performances of electrodeposited graphene oxide allowed to achieve high sensitivity, wide linear range, and a detection limit of 0.84 μM, suitable for L-Dopa detection within its therapeutic window. Interference from endogenous compounds, tested at concentrations levels typically found in drug-treated patients, was not significant. Ascorbic acid exhibited a tyrosinase inhibitory behavior and was therefore rejected from the enzymatic layer by casting an outer Nafion membrane. The proposed device was applied for L-Dopa detection in human plasma, showing good recoveries.

## 1. Introduction

Levodopa (L-Dopa or L-3,4-dihydroxyphenylalanine) is an intermediate amino acid involved in the biosynthetic pathway of endogenous catecholamines such as dopamine. It is generally produced by chemical synthesis, but it is also present in significant amounts in some plants belonging to the Fabaceae family, which could play a role as an adjuvant in the treatment of Parkinson’s disease (PD) [1]. Indeed, synthetic L-Dopa is the most effective oral dopaminergic treatment for PD, a progressive neurodegenerative disorder caused by the loss of dopaminergic neurons in the substantia nigra pars compacta [2]. Unlike dopamine, L-Dopa can cross the blood–brain barrier, and once inside the brain, it is metabolized to dopamine by aromatic amino acid decarboxylase.

L-Dopa is currently administered in combination with DOPA decarboxylase inhibitors (DDC-I), such as benserazide and carbidopa, and catechol-O-methyltransferase inhibitors (COMT-I), such as entacapone and tolcapone, in order to reduce its peripheral metabolism and increase the fraction that reaches the central nervous system [3,4]. Nevertheless, the increasing neuronal dopamine loss with PD progression leads to fluctuations in plasma L-Dopa levels, resulting in motor and non-motor complications such as dyskinesia, psychosis, and orthostatic hypotension [5]. Furthermore, the response to L-Dopa therapy shows high interpatient variability. Real-time monitoring of L-Dopa levels would be advantageous for personalized drug dosing, thus allowing for optimal therapeutic efficacy and reduction in side effects.

The instrumental methodologies currently used for L-Dopa detection [6] are mainly based on high-performance liquid chromatography (HPLC) [7,8]. Methods based on capillary zone electrophoresis are also reported [9], as well as mass spectrometry coupled to liquid chromatography [10], spectrophotometric [11], fluorescence [12], and chemiluminescence methods [13,14]. While high sensitivity and selectivity are guaranteed, disadvantages such as high costs, use of sophisticated instrumentation, complex sample pre-treatment, and long response times make such techniques not suitable for real-time monitoring of L-Dopa.

Electrochemical methods, instead, allow us to analyze complex matrices with minimal sample pre-treatment, require easy-to-use instrumentation, and provide fast response times. They also exhibit high reproducibility and sensitivity and are suitable for miniaturization [15]. This last feature is of considerable interest as it allowed, in recent years, to realize implantable devices for the in vivo monitoring of biomolecules such as glucose, lactic acid, etc. [16,17]. Having a wearable biosensor capable of continuously monitoring the plasma concentration of L-Dopa would allow for modulating the drug administration according to the severity of the motor symptoms at that moment. This would represent a real breakthrough for the management of PD.

To date, few enzyme-based biosensors are reported making use of tyrosinase for biorecognition of L-Dopa [18,19,20,21,22,23]. Among these, potentially “wearable” biosensors have been proposed made on microneedles and tested in vitro on artificial interstitial fluid [21] and bovine serum [23] and in vivo in rat abdominal cavities [23]. In order to improve selectivity towards L-Dopa, a dual sensing platform was adopted based on parallel, simultaneous, and independent enzymatic and nonenzymatic electrochemical detection. Indeed, tyrosinase (EC 1.14.18.1) exerts a double activity, catalyzing the oxidation of both monophenols (creolase or monophenolase activity) and o-diphenols (catecholase or diphenolase activity) into reactive o-quinones [24]. Creolase and catecholase activities have broad substrate specificities besides the physiological substrates, tyrosine, and L-Dopa, respectively. Endogenous neurotransmitters such as dopamine, serotonin, and adrenaline present in biological fluids or carbidopa itself, found in these samples following administration of the drug, could affect the selectivity of the enzymatic biorecognition of L-Dopa.

Although more or less effective strategies have been devised to solve the interference problems, this issue has not been properly addressed. Partial screening of potentially interferent substances has often been carried out, although tested at physiological concentrations values different from those found in plasma from PD’s patients. As an example, plasma levels of tyrosine and serotonin are lower than those in physiological conditions [25,26], whereas homocysteine plasmatic levels increase in patients under levodopa treatment [27]. L-Dopa was also mainly tested at concentrations above the therapeutic range [21,22,28].

In addition to selectivity, other critical issues arise from the examination of tyrosinase biosensors. Laborious and time-consuming fabrication protocols have been designed [19,28] to gain biosensor stability, a requirement closely related to the enzyme immobilization technique adopted. Co-crosslinking revealed a successful tool to preserve the catalytic activity of various enzymes for long periods [29,30,31]. The employment of an inert protein together with the enzyme allows to mitigate the deactivation of the catalyst and enhance the mechanical stability of the deposited layer. As far as tyrosinase immobilization, glutaraldehyde (GLU) has been employed as a reticulation agent mainly in the cross-linking mode, i.e., without the inert protein. The methods adopted often involved consecutive steps to deposit tyrosinase and GLU and were based on both dipping and drop casting. To let the tyrosinase solution air dry, the electrode was generally left at 4 °C for 24 h. An additional time ranging from 30 min up to 2 h was required for GLU deposition. When tyrosinase and GLU were both present in the deposition solution, air drying was shortened to 12 h [28]. The employment of a solution made of tyrosinase, GLU, and bovine serum albumin (BSA) as an inert protein allowed to reduce the immobilization time to 2 h [20], but the operational stability was limited to 5 days. 

The dynamic nature of drug metabolism requires continuous and long-term measurements. For this purpose, the high operational stability of the sensor is required, which, however, has sometimes been tested on very short time scales [21] or over periods extended mainly to one/two weeks [22,32]. Further progress is needed to achieve the ultimate goal of a microdevice with an integrated measurement/infusion system.

In the present work, a one-step immobilization procedure based on tyrosinase co-crosslinking through drop casting is proposed. The immobilization protocol developed allowed us to notably simplify the fabrication of the device, assuring fast execution times. A careful evaluation of the concentration ratio between the enzyme, BSA, and GLU and the compaction of the outer interface proved effective in preventing the partition and subsequent stagnation of colored compounds generated upon L-DOPA oxidation within the enzymatic membrane, thus allowing to obtain remarkable stability over time.

The effectiveness of the enzyme immobilization procedure was combined for the first time with the high reproducibility and enhanced sensitivity of a conventional glassy carbon electrode modified through the electrodeposition of graphene oxide. The response of the biosensor to typical interfering substances, tested at the concentration levels found in plasma from PD patients under pharmacological treatment, was critically evaluated. Particularly, the role of ascorbic acid as a tyrosinase inhibitor [33] was clarified. Finally, the use of an external coating of Nafion allowed the application of the sensor to the analysis of human plasma samples with satisfactory recoveries.

## 2. Results and Discussion

### 2.1. Immobilization of Tyrosinase

Biosensor performances are strictly dependent on the effectiveness of the enzyme immobilization method. In the present case, tyrosinase was immobilized by co-crosslinking [34]. To obtain an enzyme layer with high biocomponent stability and good mechanical properties, the inert protein (BSA), crosslinker (GLU), and enzyme concentrations required a careful optimization study. In Table 1, the various concentration ratios used are reported along with the sensitivity values obtained immediately after the biosensor preparation and after one and five days of usage.

Starting concentrations of 4.90 mg/mL for tyrosinase, 45 mg/mL for BSA, and 0.25% for GLU were employed, which were close to the values already optimized for the realization of a choline oxidase biosensor [29]. The resulting enzymatic layer, obtained by “casting” the solution on the surface of a glassy carbon electrode, showed a sensitivity of 6.5 ± 0.1 µA/mM in a phosphate buffer solution at pH 7.0, ionic strength 0.1 M, under rotation at 500 rpm. The protein membrane was particularly unstable, detaching from the electrode surface upon dissolution in the storage medium. The poor stability has been attributed to the relatively low GLU concentration, which is insufficient to ensure an adequate progression of the co-crosslinking reaction. GLU concentration was therefore increased to 0.5%. The resulting biosensor showed a slight improvement in stability (the enzyme layer was not released in solution) that was, however, not sufficient. The sensitivity value of 6.90 ± 0.08 µA/mM decreased by 72% after one day and by 90% after 5 days.

Given the unsatisfactory performance of the biosensor, the concentration ratio between enzyme, BSA, and GLU was further changed. As it is well known, the co-crosslinking reaction typically involves the carbonyl groups of GLU and the ε-amino groups of the L-lysine residues present on the enzyme and on the inert protein molecules, with the subsequent formation of Schiff bases. In order to preserve the enzyme catalytic activity, possible involvement of its active site in the reticulation reaction should be avoided. To mitigate such an involvement, tyrosinase concentration was decreased to 2.45 mg/mL. In this way, the active site of the enzyme molecules immobilized on the electrode surface should be preserved since the cross-linking reaction mainly occurs between GLU and the functional groups of BSA, which is present at higher concentrations. As a consequence, an increase in sensitivity could be envisaged since all the immobilized enzyme is in the active state and is, therefore, able to catalyze the substrate oxidation. Indeed, a slightly higher sensitivity value of 7.5 ± 0.1 µA/mM was recorded soon after the preparation of the biosensor. Stability, on the other hand, was not improved appreciably. A sliding of the enzymatic layer from the glassy carbon zone towards the surrounding Teflon region was visually observed, probably caused by an excessive concentration of inert protein, considerably higher than that of tyrosinase, which conferred to the protein membrane a high degree of hydration. Indeed, BSA is a natural protein with good hydration ability.

BSA concentration was then lowered to 10 mg/mL. The resulting biosensor showed a higher sensitivity of 13.5 ± 0.04 µA/mM. By lowering the inert protein concentration, the extent of enzyme crowding is reduced, and enzyme molecules are, therefore, more accessible to the substrate. Stability, however, did not improve significantly, decreasing by 50% and 70% after 1 day and 5 days from the device construction, respectively. Furthermore, as the biosensor was used, a progressive browning of the enzymatic deposit was observed. Dopaquinone, produced by the oxidation of L-Dopa, is a rather unstable molecule from which colored compounds are generated [33,35]. These compounds, through a series of enzymatic and nonenzymatic reactions, lead to the formation of the pigment melanin. 

To corroborate this explanation, the freshly prepared electrode was left immersed in the buffer solution without being used. After a few days, the enzymatic deposit was not darkened, and sensitivity was comparable with the value obtained soon after the biosensor construction. An additional step was therefore devised in the biosensor preparation aimed at compacting the outer part of the enzymatic layer. This would reduce the partition of the oxidation products, generated at the electrode/solution interface, within the enzymatic layer where their stagnation presumably affects biosensor stability. This step consisted in immersing the biosensor, immediately after its preparation, in a 0.5% glutaraldehyde solution for 15 min. The electrode was then left to air dry for approximately 30 min. 

The implementation of the fabrication protocol proved effective in preventing the membrane browning and improving biosensor stability: after 1 day of usage, sensitivity remained unchanged, and after 5 days decreased only by 4%. The long-term stability was investigated by discontinuously monitoring the biosensor sensitivity. After about three weeks, sensitivity was around 80% of the initial value. These data are very encouraging if compared with the behavior of other tyrosinase biosensors for L-Dopa detection. Indeed, response stability of a few days [20,22] or, at most, two weeks [32] is mainly reported. Some papers [21,28] did not apparently report any stability study over time, whereas stability of more than a month has been reported by Pinho et al. [19]. In the latter case, as well as in most of the proposed biosensors, laborious multi-step procedures with timescales up to or even more than 24 h were employed [18,32]. In this regard, the immobilization protocol implemented in this work is fast and easy, thus making a significant improvement. 

### 2.2. Evaluation of the Working Conditions

#### 2.2.1. Detection Potential

The operating mode of the amperometric biosensor realized in the present work is based on the catalytic oxidation of L-Dopa to dopaquinone by the immobilized tyrosinase, according to the following reaction:

tyrosinase  L-Dopa + ½ O_2_ → L-Dopaquinone + H_2_O_2_ (1)

By applying a cathodic potential to the working electrode, L-Dopaquinone is electrochemically reduced back to L-Dopa, producing a current signal proportional to the amount of L-Dopa enzymatically oxidized.

**Figure 1:**
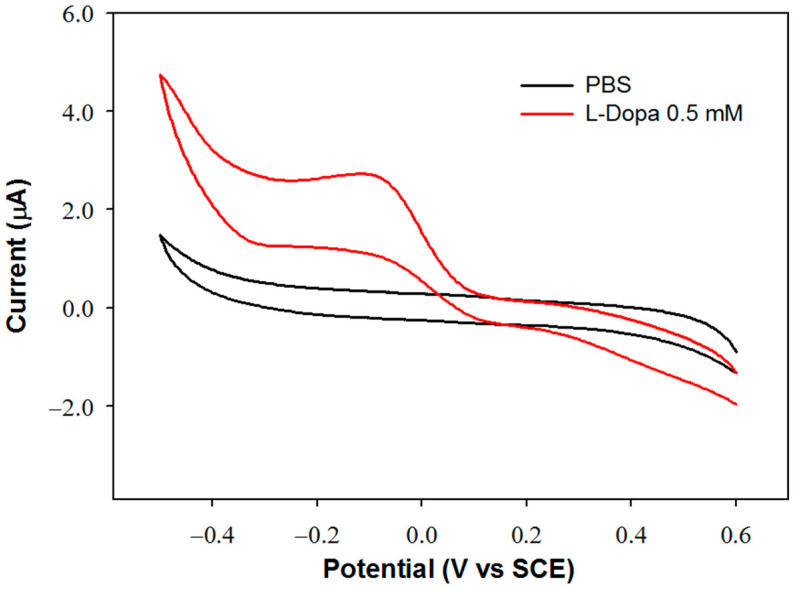
Voltammetric profile of the Glassy Carbon/Tyrosinase biosensor (GC/TYR) in phosphate buffer solution PBS (I 0.1 M, pH 7.0) (black profile) and in a 0.5 mM L-Dopa solution (red profile). Scan rate: 50 mV/s.

The detection potential for dopaquinone reduction at the GC-modified electrode was fixed by evaluating the biosensor sensitivity and linear range at different applied potentials. To select the potential values, the voltammetric profile of the biosensor was acquired in a 0.5 mM L-Dopa solution. In order to identify the reduction process of dopaquinone, the voltammetric scan was performed in the cathodic direction, and the relevant voltammogram was compared with that acquired in the buffer solution without L-Dopa. The two voltammograms are shown in Figure 1.

In the reduction scan, a peak is observed at approximately −0.1 V, attributable to the reduction of dopaquinone since it is not evident in the absence of L-Dopa. The biosensor response was then studied at this potential value and at the other two more cathodic values, −0.3 and −0.4 V. In Table 2, the parameters of the regression lines evaluated at each applied potential are reported. The sensitivity values are quite close to each other. The slightly higher value obtained by applying a detection potential of −0.4 V can be justified considering that dopaquinone at slightly acidic or neutral pH values can partially undergo cyclization, and the reduction of the cyclized form occurs at more cathodic potentials [20]. At this potential, however, a noisier current signal was observed, probably due to parallel electrode processes involving the solvent, as can be observed in the voltammogram acquired in the buffer solution. Considering that at −0.1 V, a wider linear range is obtained, and that sensitivity is only slightly lower, this value has been selected as the detection potential.

#### 2.2.2. pH

The influence of pH on the biosensor response was studied over the range of 5–7.5 by employing an acetate/phosphate buffer at a fixed ionic strength of 0.1 M, to avoid changes in the ionic composition of the supporting electrolyte. Higher pH values were not tested since, as it is well known, L-Dopa is unstable in alkaline solutions and naturally degrades over time [1]. 

**Figure 2:**
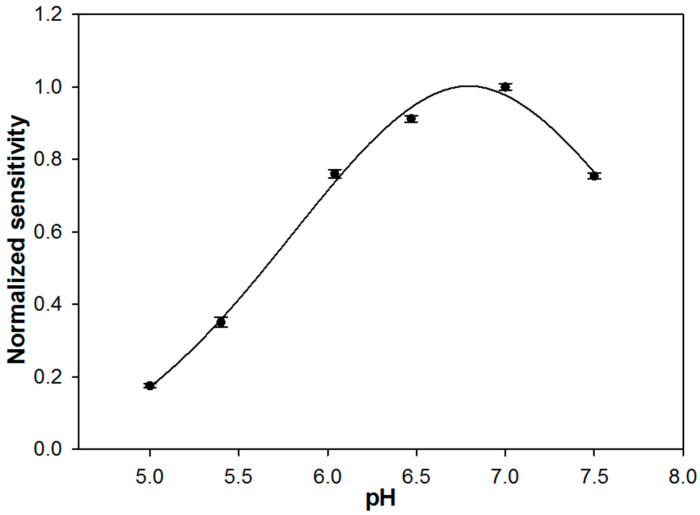
Normalized sensitivities of a typical rotating disk GC/TYR biosensor for L-Dopa as a function of pH. Error bars indicate the relevant standard deviations coming from responses derived from three different measurements. Supporting electrolyte: acetate/phosphate buffer (I = 0.1 M).

In Figure 2, the sensitivity values of the biosensor, derived from the slope of the calibration lines, are reported as a function of pH. A bell-shaped curve was obtained with a maximum between pH 6.5 and 7, in agreement with the optimal pH of 6.8 reported for native enzymes [36]. As it is known, enzymatic immobilization could cause a shift in the optimal pH value [37] that, in the present case, was not observed. The decrease in sensitivity observed for pH values lower and higher than 6.8 could be due to the denaturation of the biocomponent or to the formation of improper ionic forms of the active site of the enzyme and of the substrate. The ionizable amino acid residues surrounding the catalytic site of an enzyme are able to give electrostatic interaction with the substrate only if they are both in the proper ionic form. To evaluate which is the most plausible explanation, the calibration curves were acquired sequentially from pH 7.5 to pH 5 and then again at pH 7. The sensitivity value recorded at pH 7 did not change significantly, suggesting that a possible decrease in enzyme stability is to be excluded. 

A pH of 7.0 was selected since it assures maximum enzyme activity and is close to the physiological value.

### 2.3. Analytical Performances of the Biosensor GC/TYR

**Figure 3:**
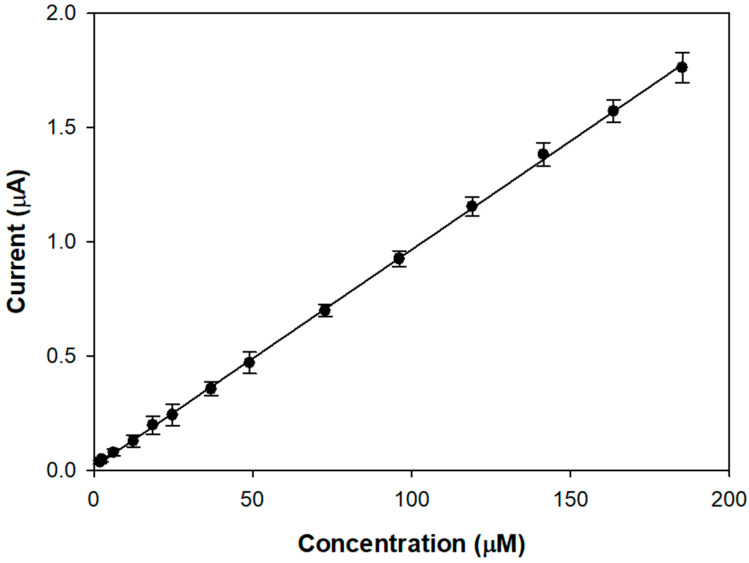
Linear range relevant to successive additions of a L-Dopa standard solution for a typical GC/TYR biosensor. Rotation rate: 500 rpm. Supporting electrolyte: phosphate buffer solution pH 7.0, I 0.1 M. Each experimental data represents the mean of triplicate measurements; the error bars indicate the relevant standard deviations.

Figure 3 shows the linear range of a typical calibration curve of the modified electrode acquired upon the addition of aliquots of a stock L-Dopa solution in an air-saturated buffer. 

The proposed biosensor allows achieving linear L-DOPA responses from 1 up to 190 µM, which seems better than many tyrosinase devices (0–20 µM [23], 5–30 µM [22], 0.8–22.3 µM [20]). The presence of the compact outer layer slows down the diffusion of the analyte and its oxidation product, increasing linearity. From the slope of the linear regression line, a sensitivity value of 9.51 ± 0.04 µA/mM (R^2^ = 0.9997) was calculated, which is satisfactory considering that a conventional glassy carbon electrode was used. 

The reproducibility of the biosensor was investigated using the sensitivity values derived from the linear ranges. For three electrodes prepared in the same way, an acceptable RSD of 8.2% was obtained, indicating good electrode-to-electrode reproducibility of the fabrication method. Using the same electrode, the within-day (n = 3) and day-to-day (n = 5) relative standard deviations for replicate measurements of sensitivity were 4.5% and 6.7%, respectively.

The precision of the proposed device is almost comparable with other tyrosinase biosensors. In some cases, better values were achieved [21], but the evaluation was made on a short time scale, using a fixed L-Dopa concentration and not the sensitivity values derived over the whole linear concentration range.

### 2.4. Selectivity of the Biosensor

To evaluate the selectivity of the biosensor, the response to potential interfering sub-stances at the concentration levels found in plasma samples from PD patients under pharmacological treatment was investigated. The concentration of L-Dopa, carbidopa, and dopamine solutions was set at 2.5 µM, 0.2 µM, and 0.32 µM, respectively, which are the mean values normally found in plasma approximately one hour after the oral intake of a single dose of Stalevo^®^ tablet formulation (100 mg of levodopa, 25 mg of carbidopa, and 200 mg of entacapone) [38]. The therapeutic range reported for L-DOPA is 500–1600 ng/mL [38,39].

Tyrosine, serotonin, homocysteine, and ascorbic acid were also considered. Tyrosine is one of the physiological substrates of tyrosinase that could represent a source of interference for the determination of L-DOPA in biological fluids. In individuals with PD, plasma levels of tyrosine are lower than in physiological conditions [25]. Since precise data were not available, tyrosine was tested at the same concentration value of L-Dopa. Serotonin, a neurotransmitter involved in several physiological functions (sleep–wake, mood, etc.), was investigated at the concentration value of 0.15 µM reported for PD patients [26]. Higher plasma values are found in healthy subjects. 

On the other hand, PD patients under treatment with L-Dopa exhibit higher homo-cysteine plasmatic levels [27]. The upper limit for healthy subjects is 15 µM; above this value, hyperhomocysteinemia occurs, a risk factor for serious medical conditions. In this case, detailed values were also not found, and then a concentration of 20 µM was selected, higher than the upper limit. Finally, ascorbic acid was studied at a concentration of 100 µM [40].

**Figure 4:**
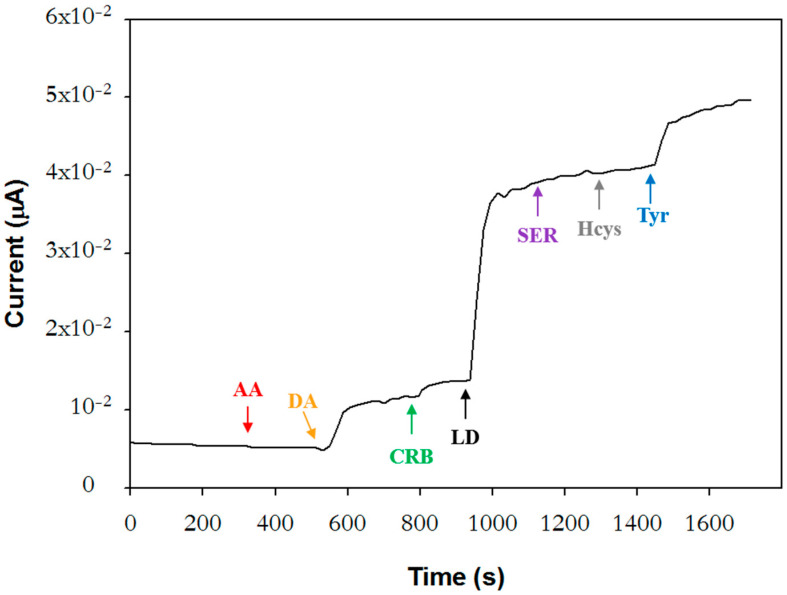
Chronoamperogram acquired in phosphate buffer solution (pH 7.0, I 0.1 M) upon successive injections of the following compounds: 100 μM ascorbic acid (AA), 0.32 µM dopamine (DA), 0.2 μM carbidopa (CRD), 2.5 μM L-Dopa (LD), 0.15 μM serotonin (SER), 20 µM homocysteine (Hcys), and 2.5 μM tyrosine (Tyr). Rotation rate: 500 rpm.

Figure 4 shows the current profile acquired upon successive injections of interferents and L-Dopa. The interference from all the investigated compounds is negligible except for dopamine and tyrosine, which contribute respectively to 20% and 22% of the L-Dopa signal. It is worth noting that the dopamine values normally tested with other tyrosinase biosensors [22] fall within the range 5.27 × 10^−11^–7.91 × 10^−10^ M reported for healthy individuals [18] and are, therefore, notably lower than the concentration employed in the present work. Furthermore, L-Dopa was often tested at levels higher than the value used in this work, above the therapeutic range [21].

When the experiment of Figure 4 was repeated by randomly injecting the interfering species in a different order, ascorbic acid caused a significant decrease in the cathodic current, as it is possible to see in Figure S1. A plausible explanation could be that dopaquinone-like products, generated upon the tyrosinase interaction with its substrates previously injected, are chemically reduced by ascorbic acid. Accordingly, if ascorbic acid is injected before tyrosinase substrates, this behavior is not observed. As already described in the introductory part, ascorbic acid is reported to be a tyrosinase inhibitor [41]. Indeed, its role as an inhibitor or inactivator has been critically discussed. As an antioxidant, reducing back o-dopaquinone to L-DOPA, thus avoiding dopachrome and melanin formation, it has been considered a melanogenesis inhibitor rather than a “true tyrosinase inhibitor” [33]. However, in aerobic conditions, a suicide inactivation mechanism from ascorbic acid has been described, which was attributed to the enzymatic form of oxytyrosinase [42].

In order to overcome this drawback, the biosensor was covered with an outer layer of Nafion, a polymer widely used as a suitable and biocompatible protective layer or as an enzyme-entrapping membrane [20,43]. At pH 7, Nafion is negatively charged and is, therefore, able to reject ascorbic acid by electrostatic repulsion, a more specific exclusion mechanism than that of the widely used insulating polymers. In the latter case, a selective partition/permeation of differently charged compounds occurs, thanks to the compact structure of these films, free from defects and pinholes [44,45]. 

The Nafion coating was deposited on the biosensor by drop casting 4 µL of a 1% solution. Injecting L-Dopa and then ascorbic acid at the same concentration values used before, no decrease in the current signal was observed anymore. The Nafion coating, however, caused a marked attenuation of the L-Dopa current response as well, affecting the biosensor sensitivity. 

The underlying GC electrode was then modified by electrodeposition of a graphene oxide layer before enzyme immobilization to enhance the surface area and assure higher current responses toward L-Dopa.

### 2.5. Electrodeposition of Graphene Oxide

Graphene oxide (GO) is deposited on conventional electrode surfaces mainly by drop casting due to its fast and easy execution. This technique is characterized by poor reproducibility and difficulty in controlling the thickness of the deposited layer. Conversely, electrochemical deposition is a reproducible technique suitable for any shaped and sized electrode. It is also “eco-friendly” since it is based on the employment of aqueous solutions. Unlike drop casting, it is rarely used for the realization of modified electrodes based on graphene and its derivatives [46].

**Figure 5:**
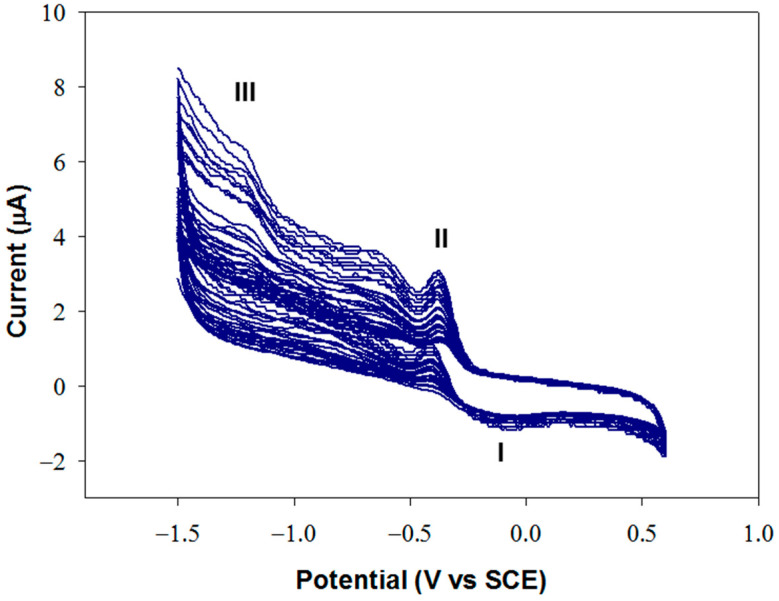
Voltammetric profile relevant to the electrochemical deposition of GO, from a 0.1 mg/mL suspension in carbonate buffer solution at pH 9.0, I 0.1 M, by scanning the potential in the range −1.5/0.6 V vs. SCE for 30 cycles. Scan rate: 50 mV/s.

An optimization study of the electrochemical deposition of graphene oxide was carried out to ensure high detection performances. A GO suspension with a concentration of 0.1 mg/mL in carbonate buffer at pH 9.0, I 0.1 M was employed, and potential was scanned in the range −1.5/+ 0.6 V at 50 mV/s. Figure 5 shows a typical growth profile on glassy carbon. 

At pH 9, GO is negatively charged and is drawn close to the electrode surface during the application of positive potentials. When the potential becomes sufficiently cathodic, reduction occurs, causing an irreversible agglomeration of the GO sheets, which precipitate on the adjacent electrode surface. The voltammogram reported in Figure 5 shows an anodic peak (I) at approximately −0.1 V and two cathodic peaks approximately at −0.37 V (II) and −1.25 V (III). The increase in the intensity of these peaks, in particular of peak III, indicates that GO is being deposited on the electrode. Peak III was attributed to the irreversible electrochemical reduction of GO, while peaks I and II were assigned to the redox behavior of the oxygen-containing functional groups present on the planar graphene sheet [46].

The optimization of the number of scanning cycles was performed using a solution of 5 mM potassium ferricyanide in 0.1 M KNO_3_ as an electrochemical probe. For each fixed number of cycles, the voltammetric profile of ferricyanide was acquired immediately after the deposition and over time to verify the stability of the resulting GO layer. Figure S2 shows the overlap of the voltammetric profiles recorded at the different growth cycles. As expected, the current increases progressively with an increasing number of cycles, indicating increased electrochemical active sites [46]. The growing reduced graphene oxide could produce a wrinkled texture on the electrode surface, which increased the superficial area, as was demonstrated through morphological characterization by SEM images [47]. From 40 cycles, however, the deposit was too thick to spread on the Teflon region and exhibited poor mechanical stability, evidenced by the marked attenuation of the voltammetric profile already after one hour. Electrode modification using 10 and 20 cycles did not allow for a significant increase in ferricyanide current. The number of cycles was therefore set at 30 as the right compromise between high response and adequate stability (the ferricyanide profile did not change significantly even after one day, as shown in Figure S3).

The concentration of the deposition solution was then changed by increasing it to 0.25 mg/mL. No improvements were detected in terms of sensitivity or stability, probably because a less efficient dispersion of the GO suspension occurred at higher concentrations.

The optimized deposition procedure showed high reproducibility. A relative standard deviation of 3% (n = 3) was evaluated on the cathodic peak current of the ferricyanide voltammetric profile acquired on freshly prepared GC/GO electrodes, confirming the enhanced performances of electrochemical deposition. 

The electrode surface area was calculated from the slope of the Randles–Sevcick equation. The cathodic peak currents in the voltammetric profile of ferricyanide were measured by varying scan rates. The GO layer allowed to increase the surface area of the electrode by 80% with respect to bare GC.

### 2.6. Characterization of the GC/GO/Tyrosinase Biosensor

Given the overall high performance, the modified GC/GO electrode was used for the construction of the tyrosinase biosensor, employing the immobilization protocol previously optimized. A tyrosinase biosensor for L-Dopa detection realized by electrodepositing GO has never been proposed before. Xiao et al. [48] realized a wearable electrochemical sensor for levodopa quantification in sweat based on a metal–organic framework/graphene oxide composite deposited by drop casting. 

**Figure 6:**
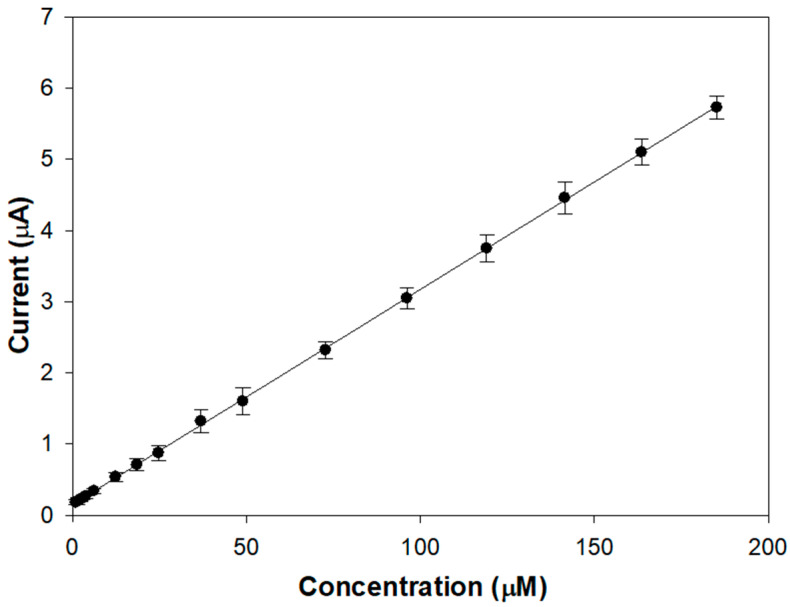
Linear range of the calibration curve for a typical GC/GO/TYR biosensor. Rotation rate: 500 rpm. Supporting electrolyte: phosphate buffer solution pH 7.0, I 0.1 M. Each experimental data represents the mean of triplicate measurements; the error bars indicate the relevant standard deviations.

In Figure 6, the linear range of the calibration curve realized using the same experimental conditions as Figure 3 is reported. From the slope of the regression line, a sensitivity of (30.2 ± 0.1) µA/mM (R^2^ = 0.9998) was evaluated, which is significantly increased. This finding is promising from the perspective of depositing the Nafion outer layer, which is necessary to ensure selectivity without excessively attenuating the response to L-Dopa.

**Figure 7:**
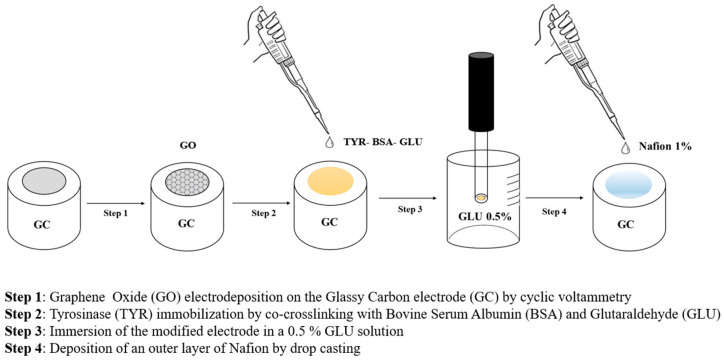
Schematic representation of the steps used for the realization of the biosensor.

Nafion was then deposited by drop casting following the same procedure previously employed. In Figure 7, all the steps employed for preparing the biosensor GC/GO/TYR/Nafion are schematized. The presence of the Nafion coating resulted in a slight increase in the linear range up to approximately 210 µM (Figure S4). Sensitivity, as to be expected, decreased to 3.21 ± 0.01 µA/mM (R^2^ = 0.9996) but is still appreciable. From the regression line, a detection limit of 0.84 µM was calculated [49], which is suitable for detecting L-Dopa in plasma samples from PD patients, testifying to the validity of the device herein developed in the scenario of tyrosinase biosensors already proposed for L-Dopa detection. 

Modified electrodes based on nanostructured materials combined with tyrosinase with detection limits comparable to or even higher have been realized. To cite some examples, in the case of a biosensor based on gold nanodendrites, a detection limit of 1.25 µM was obtained [28], while in the case of a biosensor based on carbon nanotubes, the value was 2.5 µM [20]. Elsewhere, biosensors with LODs significantly lower, down to 0.18 µM [32] or even 1 nM [19], were realized, requiring, however, an extremely complex procedure for sensor preparation. Conversely, in this work, we propose an easy and efficient procedure for realizing the overall device within a short time frame. Moreover, it should be considered that L-Dopa plasmatic levels in PD patients do not require such low LODs. 

### 2.7. Real Sample Analysis

To evaluate the reliability of the proposed biosensor in practical applications, it was employed for L-Dopa detection in human plasma. Plasma is the biological fluid generally investigated to check the efficacy of drug administration. Alternative biological fluids have also been explored. An integrated sweatband sensor on a wearable platform, capable of monitoring dynamic changes in L-Dopa in sweat, has been developed as a less invasive device [28]. L-Dopa is excreted in sweat at concentration values correlated with those present in human plasma. Stationary iontophoretic stimulation and physical exercise were used to extract sweat. As an alternative to vigorous sweat stimulation methods, a biosensor has been proposed that exploits natural perspiration on the fingertips, favored by the high density of eccrine sweat glands [22]. In this case, the dynamic profile of L-Dopa in sweat after drug administration was monitored in semi-continuous mode.

In this work, a plasma sample from a healthy subject was tested since plasma from PD patients was not available. This sample, prior to analysis, was not subjected to any pre-treatment. The experiments were performed in triplicate, and recovery tests were conducted via the standard addition method. The results are illustrated in Table 3. The obtained recoveries ranged from 90.8% to 102.4%, thereby validating the accuracy and practicability of the developed biosensor.

## 3. Materials and Methods

### 3.1. Reagents and Sample

Tyrosinase (EC 1.14.18.1 from *Agaricus bisporus*, ≥1000 unit/mg solid), glutaraldehyde (grade II, 25% aqueous solution), bovine serum albumin (BSA, 98%), 3,4-dihydroxy-Lphenylalanine (L-Dopa ≥ 98%), dopamine (99%), tyrosine (99%), ascorbic acid (99%), serotonin (98%), homocysteine, carbidopa, Nafion 110 W, graphene oxide (4 mg/mL), potassium chloride (99%), nitric acid (70%, d = 1.413 g/mL), acetic acid (99.7%, d = 1.049 g/mL), sodium hydroxide (98%), potassium nitrate (99%), sodium bicarbonate (99%), monopotassium phosphate (99%), and potassium ferrocyanide (99%), were purchased from Sigma-Aldrich. Ethanol (≥99%) was purchased from Fluka. Powdered alumina was supplied by Buehler (Micropolish II 0.05 µm, deagglomerated gamma alumina). The above chemicals were used without further purification. 

Ultra-pure water used in all the experiments was obtained from a combined Elix-5/Milli-Q system (Millipore, S.p.A. Milan, Italy). 

The interfering solutions were freshly prepared. L-Dopa standard solutions were stored at +4 °C for no more than 2 days. A healthy adult volunteer provided the plasma sample, which was injected directly into the electrochemical cell without any pre-treatment.

### 3.2. Apparatus

A 263A potentiostat/galvanostat (EG&G Princeton Applied Research, Princeton, NJ, USA) was employed to carry out cyclic voltammetry and chronoamperometry experiments. Data were acquired by using the M270 software version 4.23 (EG&G). A conventional three-electrode cell was used consisting of a saturated calomel reference electrode (SCE), a platinum counter electrode, and a working electrode made of a glassy carbon disk (2.5 mm diameter) inserted in a PTFE body. 

Rotating disk electrode (RDE) experiments were performed by a CTV101 Speed Control Unit, EDI 101 Rotating Disc Electrode (Radiometer Copenhagen). A rotation rate of 500 rpm was adopted. 

### 3.3. Biosensor Preparation

Prior to electrode modification, the GCE surface was cleaned following an optimized procedure. The electrode was mechanically polished with abrasion by alumina (0.05 µm particles) and sequentially sonicated firstly in double distilled water and ethanol (*v*/*v* 1:1) for 2 min, then in double distilled water and nitric acid (*v*/*v* 1:1) for 2 min and finally in double distilled water for 2 min. Between each sonication, the electrode was rinsed with pure water. 

Then the GCE was covered with electrochemically deposited graphene oxide. The electrochemical deposition was performed using a 0.1 mg/mL GO suspension, prepared in a carbonate buffer solution (pH 9.0, ionic concentration 0.1 M), and ultrasonicated for 2 h. Before starting, the suspension was bubbled with nitrogen for 30 min. The electrochemical deposition was performed by cyclic voltammetry in the potential range −1.5 V/+ 0.6 V vs. SCE at the scan rate of 50 mV/s for 30 cycles. After electrodeposition, the modified electrode was gently washed with double distilled water and air-dried at room temperature before enzyme immobilization.

Tyrosinase immobilization was carried out by mixing 5 µL of a 19.6 mg/mL tyrosinase solution prepared in a phosphate buffer solution (PBS) at pH 7.0, I 0.1 M, 20 µL of PBS, 5 µL of an 80 mg/mL BSA solution, prepared in the same buffer, and 10 µL of a 2% GLU solution (25% GLU solution diluted with phosphate buffer). Further, 4 µL of the resulting solution was cast onto the surface of the modified glassy carbon electrode, avoiding air bubble formation, and left to dry at room temperature for 20 min. Successively, the biosensor was dipped in a 0.5% glutaraldehyde solution for 15 min and then air-dried at room temperature for 30 min. 

For application in plasma samples, 4 µL of a 1% Nafion solution (5% Nafion solution diluted with water and ethanol (*v*/*v* 3:1)) was cast onto the biosensor and left to dry at room temperature for 90 min. The biosensor, when not used, was stored in PBS at +4 °C.

### 3.4. Electrochemical Measurements

Electrochemical measurements were performed by applying a detection potential of −0.1 V versus SCE unless otherwise stated. Aliquots of standard solutions of L-DOPA and interfering compounds were injected into the electrochemical cell containing 20 mL of phosphate buffer solution, pH 7, ionic concentration 0.1 M. Rotation rate was 500 rpm. Air-saturated solutions were employed at room temperature.

## 4. Conclusions

A novel amperometric biosensor for L-DOPA detection has been proposed based on tyrosinase immobilized by co-crosslinking on a glassy carbon electrode previously modified with graphene oxide.

A careful evaluation of the experimental conditions to use for enzyme deposition proved capable of ensuring, through a fast and easy-to-execute protocol, a notable gain in stability and satisfactory precision. The electrochemical deposition of graphene oxide was also optimized, allowing it to realize highly reproducible modifications and to improve the analytical performances in terms of sensitivity and limit of detection. Such an improvement was revealed successful when a Nafion coating was cast on the modified electrode to solve interference problems. In this regard, endogenous compounds naturally occurring in human plasma were tested at the concentration levels found in PD patients under drug administration. The evaluation of the actual concentration ratio between L-DOPA and potential interferents is a generally overlooked issue that has been received in this work the required consideration. In particular, the interferent behavior of ascorbic acid as a tyrosinase inhibitor was clarified, never evidenced in previous studies on biosensors selectivity. The reliability of the device in practical applications was demonstrated by analyzing a human plasma sample of a healthy subject. Good recovery percentages were achieved. 

The overall performances of the biosensor and the fast and easy procedure designed for its preparation, which constitutes a significant advancement in the field of tyrosinase biosensors, encourage its future employment for L-DOPA quantification in plasma samples from PD patients. The evaluation of levodopa plasmatic fluctuations is of interest for monitoring its therapeutic efficacy. 

## Figures and Tables

**Table 1:** Concentration values of Tyrosinase, BSA, and GLU used to realize the various biosensors and relative decrease in sensitivity over time.

Biosensor	Tyrosinase mg/mL	BSA mg/mL	GLU % (*v*/*v*)	Sensitivity ^1^ µA/mM	% Decrease after 2 Days	% Decrease after 5 Days
1	4.90	45	0.25	6.5 ± 0.1	n.d. ^2^	n.d. ^2^
2	4.90	45	0.50	6.90 ± 0.08	72	90
3	2.45	45	0.50	7.5 ± 0.1	70	86
4	2.45	10	0.50	13.50 ± 0.04	50	70
5 ^3^	2.45	10	0.50	8.0 ± 0.1	0	4

^1^ Sensitivity of fresh prepared biosensors. ^2^ Not detected due to enzyme layer detachment. ^3^ Biosensor with the outer protective layer realized by immersion in a 0.5% GLU solution for 15 min.

**Table 2:** Parameters of the regression lines acquired at the different detection potentials.

Applied Potential (V vs. SCE)	Sensitivity (µA/mM)	R^2^	Linear Range (µA)
−0.1	7.19 ± 0.06	0.9993	up to 190
−0.3	7.1 ± 0.1	0.9983	up to 130
−0.4	7.7 ± 0.2	0.9976	up to 110

**Table 3:** Determination of L-Dopa in a human plasma sample.

Added (μM)	Found (μM) (mean ± SD, n = 3)	Recovery %
0	n.d. ^1^	-
1.25	1.28 ± 0.34	102.4
2.50	2.27 ± 0.15	90.8

^1^ Not detected.

## Data Availability

Data are available on request due to privacy restrictions.

## Supplementary Materials

The following supporting information can be downloaded at: https://www.mdpi.com/article/10.3390/molecules28135239/s1, Figure S1: Chronoamperogram acquired in phosphate buffer solution (pH 7.0, I 0.1 M) upon successive injections of the following compounds: 2.5 μM L-Dopa (LD), 0.32 µM dopamine (DA), 0.2 μM carbidopa (CRD), 2.5 μM tyrosine (Tyr), 100 μM ascorbic acid (AA), 20 µM homocysteine (HCY) and 0.15 μM serotonin (SER). Rotation rate: 500 rpm; Figure S2: Cyclic voltammograms relevant to 5 mM potassium ferricyanide in 0.1 M KNO_3_ acquired on GC electrode (black profile) and on GC electrode modified with graphene oxide (GC/GO) grown for 10, 20, 30, 40 and 50 cycles. Scan rate: 50 mV/s; Figure S3: Cyclic voltammograms relevant to 5 mM potassium ferricyanide in 0.1 M KNO_3_ acquired on GC electrode (yellow profile) and on GC electrode modified with graphene oxide (GC/GO) grown for 30 cycles soon after its deposition (black profile), after 1 h from the deposition (red profile) and after one night from the deposition (green profile). Scan rate: 50 mV/s; Figure S4: Linear range of the calibration curve for a typical GC/GO/TYR/Nafion biosensor. Rotation rate: 500 rpm. Supporting electrolyte: phosphate buffer solution pH 7.0, I 0.1 M.
