# Supplementary material for: An Amperometric Biosensor Based on a Bilayer of Electrodeposited Graphene Oxide and Co-Crosslinked Tyrosinase for L-Dopa Detection in Untreated Human Plasma
Source: Molecules. 2023 Jul 6;28(13):5239. doi: 10.3390/molecules28135239 (PMC10343914; doi:10.3390/molecules28135239)
Supplement: Supplementary file 1 [file molecules-28-05239-s001.zip › molecules-2468120-supplementary.pdf]

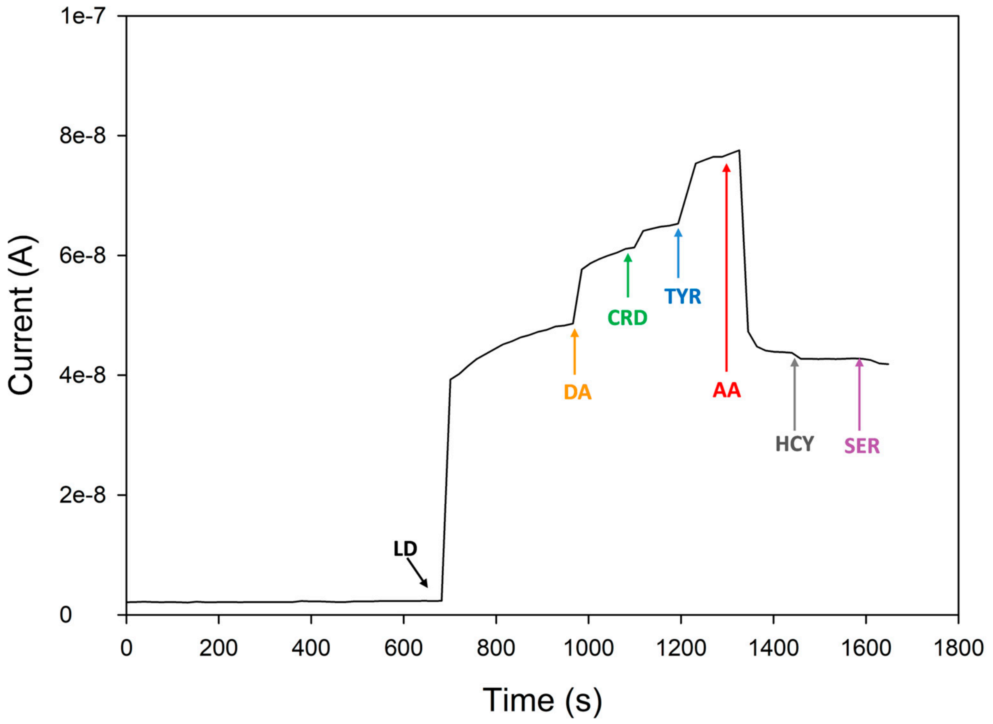

Figure S1. Chronoamperogram acquired in phosphate buffer solution (pH 7.0, I 0.1 M) upon successive injections of the following compounds: 2.5 μM L-Dopa (LD), 0.32 μM dopamine (DA), 0.2 μM carbidopa (CRD), 2.5 μM tyrosine (Tyr), 100 μM ascorbic acid (AA), 20 μM homocysteine (HCY) and 0.15 μM serotonin (SER). Rotation rate: 500 rpm.

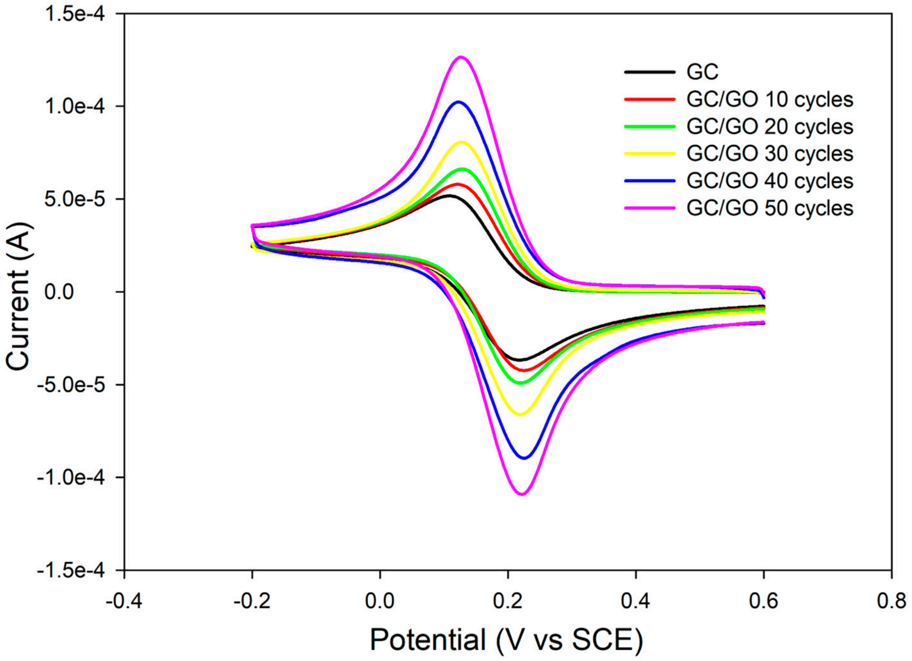

Figure S2. Cyclic voltammograms relevant to 5 mM potassium ferricyanide in 0.1 M KNO_3 acquired on GC electrode (black profile) and on GC electrode modified with graphene oxide (GC/GO) grown for 10, 20, 30, 40 and 50 cycles. Scan rate: 50mV/s.

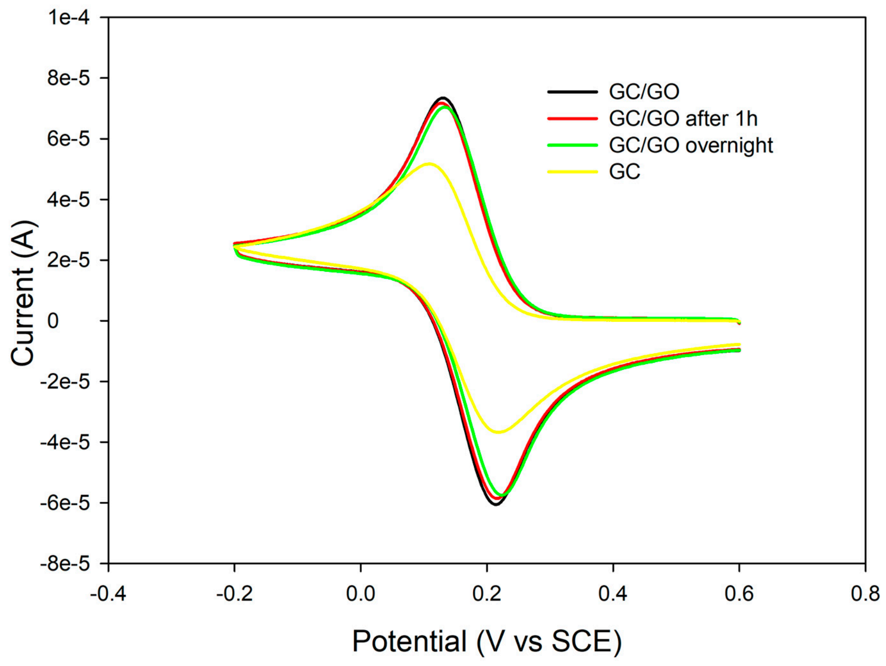

Figure S3. Cyclic voltammograms relevant to 5 mM potassium ferricyanide in 0.1 M KNO_3 acquired on GC electrode (yellow profile) and on GC electrode modified with graphene oxide (GC/GO) grown for 30 cycles soon after its deposition (black profile), after 1h from the deposition (red profile) and after one night from the deposition (green profile). Scan rate: 50mV/s.

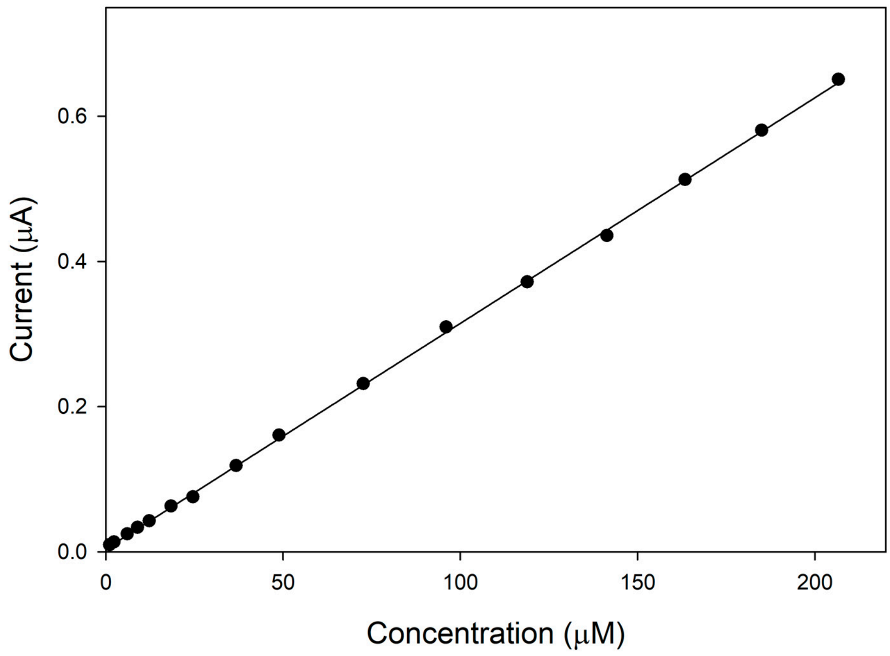

Figure S4. Linear range of the calibration curve for a typical GC/GO/TYR/Nafion biosensor. Rotation rate: 500 rpm. Supporting electrolyte: phosphate buffer solution pH 7.0, I 0.1 M.
